# Demonstrating the principal mechanism of action of medical devices intended for vaginal use on reconstructed human vaginal epithelium: the case of two hyaluronic acid-containing devices

**DOI:** 10.3389/fdsfr.2024.1445519

**Published:** 2024-09-17

**Authors:** Marisa Meloni, Barbara De Servi, Francesco Carriero, Emmanuelle Simon O’Brien, Dounia Houamel, Philippe Deruelle, Vincent Castagné

**Affiliations:** ^1^ VitroScreen, In Vitro Research Laboratory, Milan, Italy; ^2^ Biocodex SA, Centre de R&D, Compiègne, France; ^3^ Department of Obstetrics and Gynecology, Montpellier University Hospital, Montpellier, France

**Keywords:** film forming, medical device regulation, 3D human tissues, re-epithelization, vaginal dryness

## Abstract

Regulation (EU) 2017/745 on medical devices (MDR) has significantly modified the rules to be adopted for MD qualifications and classification. New requirements require robust evidence on mechanisms of action (MoAs) that cannot be produced by existing common EU or ISO standards. Therefore, on a “case-by-case basis,” a new evidence-based non-clinical approach to MD qualification must be defined. In this study, an *in vitro* experimental approach is described to assess the physicochemical and mechanical MoA of two hyaluronic acid (HA)-based medical devices: Mucogyne^®^ Gel and Mucogyne^®^ Ovule for vaginal use. They both act as moisturizers and lubricants as well as a healing adjuvant by promoting the continued moisture of the vulvovaginal area. The MoA of these two products has been demonstrated by using a 3D reconstructed human vaginal epithelium (HVE) model in a homeostatic physiological state and in stressed conditions. Film forming and persistency properties were assessed on intact HVE tissues by caffeine permeation assay and Lucifer Yellow (LY) localization on HVE vertical sections. Healing properties were assessed on injured HVE tissues by trans-electrical epithelial resistance (TEER) measurements associated with histo-morphological analysis (H&E), and moisturizing efficacy was evaluated on HVE tissues cultured in dry conditions by histomorphological analysis (H&E) and aquaporin 3 (AQP3) expression and localization by immunohistochemistry (IHC). Using the same “dry” HVE model, the non-pharmacological action of the two products was addressed by CD44 (hyaluronic acid receptor) expression and localization. The results suggest that *in vitro* evaluations can provide robust results on a human-relevant experimental model for the intended use of the products and supports clinical data with mechanistic information which may not be achieved with *in vivo* studies but are particularly important for product qualification. The results also underline the specific relative efficacy of the mechanisms investigated for Mucogyne^®^ Gel and Mucogyne^®^ Ovule in line with their different formulation types (respectively, hydrophilic and lipophilic) that influence the action of the active ingredient HA. The present *in vitro* non-clinical evaluation of HVE combined with clinical investigation data obtained in women explain why Mucogyne MDs provide significant benefits in various physiological or pathological situations, including vaginal dryness and healing.

## 1 Introduction

In gynecology, genitourinary tract alterations associated with vulvovaginal atrophy (VVA) represent the most striking and acknowledged manifestation of menopause, being found in at least 50% of menopausal women (Palma et al., 2016; Pérez-López et al., 2021a). The main bothersome symptoms of VVA include vaginal dryness, redness, itching, pain at intercourse, and reduced lubrification; these can affect women’s quality of life and can get worse if not treated. The therapeutic management of VVA should follow a sequential order, considering a woman’s age, symptoms, general health, and treatment preference. Treatments include both local and systemic hormonal (Minkin and Reiter, 2014; Costa et al., 2021) or non-hormonal options (Naumova and Castelo-Branco, 2018; Pérez-López et al., 2021b; Benini et al., 2022). In certain cases, non-pharmacological treatments are beneficial and are considered the first-line therapy for women with contraindications or fear of hormonal treatments. These include moisturizers and lubricants (De Seta et al., 2021; Gao et al., 2021; Potter and Panay, 2021).

Vaginal dryness can occur at any age, with prevalence ranging from 13% to 31%, and is primarily linked to reduced estrogen levels during breastfeeding, menopausal transition, premature ovarian failure, oophorectomy, or pelvic radiotherapy. Other medical conditions can also induce vaginal dryness, including untreated hypertension, diabetes, metabolic syndrome, pituitary disorders, neuropathies, and dermatoses. Some medications, including antihistamines, decongestants, antidepressants, antiestrogen therapy, chemotherapy, diuretics, and progesterone-predominant oral contraceptives, may also provoke vaginal dryness. Additionally, the excessive use of showers, hot baths, detergents and dehydrating soaps, highly absorptive tampons, and male condoms with insufficient external lubricant are also recognized as risk factors for vaginal dryness, which is thus a concern for all women (Wilhite, 2018).

Regulation (EU) 2017/745 on medical devices (MDR) has assigned a regulatory status to those products, composed of substances or combinations of substances formerly indicated as “borderline products” by providing an *ad hoc* classification rule (Rule 21) and requirements (e.g., Annex I, requirements regarding design and manufacture, point 12.2; Annex II, point 6.2. Additional information required in specific cases) (Marletta, 2020). However, it remains fundamental that manufacturers who produce substance-based medical devices (SBMDs) respond to MDR requirements and provide sound scientific evidence on the MD principal mode of action reach conclusions about its qualification. It is expected that a MD achieves its principal intended action by mechanical, chemical, or physical means and that it cannot principally act by pharmacological, immunological, or metabolic (Ph.I.M.) actions which are reserved for medicinal products. Different approaches to support the principal MoA for product qualification are 1) literature research, 2) pharmaco-toxicological expertise on functional substances, 3) experimental non-clinical studies, and 4) clinical data (including post-marketing surveillance data). They can be used separately or in a complementary way: however, the quality, biological relevance, and robustness of the scientific data produced remains essential to accepting/including one or other type of data in the biological evaluation.

Clinical studies can hardly demonstrate the MoA but rather confirm it with clinical evidence according to the intended use of the product, the target population, and realistic exposure. SBMD qualification by sound experimental data can thus rely on *in vitro* models reaching human predictivity by using human cells based and biologically relevant test systems. Multiple advantages over simplified 2D bidimensional cell culture are provided by 3D human reconstructed tissues that closely mirror morphological and functional characteristics of the human tissues (Pellevoisin et al., 2018a; Pridgeon et al., 2018). For these reasons, they represent a closer alternative with biological relevance and predictivity to humans. This ethical and evidence-based approach to MD testing has only recently begun; so far, few studies describe protocols to assess the MoA of MD in different therapeutic areas—the gastrointestinal tract (De Servi et al., 2016; Sardi et al., 2018; Pellegatta et al., 2020; Meloni et al., 2021; Ceriotti et al., 2022) dermatology (Casiraghi et al., 2017), gynecology (Stabile et al., 2021), and the respiratory tract ((Huang et al., 2019; De Servi et al., 2020). Additionally, 3D models have been used to assess local tolerance for gynecological products (Ayehunie et al., 2011; Costin et al., 2011; Ayehunie et al., 2018); one of them, reconstructed human epidermis (RHE), is now accepted as ISO 10993-23 for the biological evaluation of MD, their materials, and extracts (Pellevoisin et al., 2018b; De Jong et al., 2018; Kandárová et al., 2018). Few others have argued the concept of pharmacological MoA with respect to the definitions reported in MEDDEV 2.1/3 rev 3 and in the more recent MDCG 2022-05 on borderline products between MD and medicinal products under Directive 2001/83/EC and MDR, respectively, with the aim of making this definition clear and useful (Racchi et al., 2016; Racchi and Govoni, 2020; Leone, 2022).

The aim of the present research was to demonstrate that it was possible to assess the principal mechanism of action (that must be based on mechanical-physico-chemical means) of two Class IIb SBMDs according to MDR requirements without using animal models by a non-clinical ethical *in vitro* experimental approach. The intimate gel Mucogyne^®^ Gel and vaginal Mucogyne^®^ Ovule, produced by Biocodex SA (France), both contain hyaluronic acid (HA) with a liposome structure in the gel formulation, and they act by maintaining the physiological moisture of the vulvovaginal mucosa, compensating by a lubricant action for any natural secretion deficiencies. Sodium hyaluronate, a water soluble molecule, is the main active ingredient in both MDs (at 0.1% in the gel and 0.22% in the ovule), but the chemical environment on which it acts is different: the gel a being a hydrophilic formulation and the ovule lipophilic, it is possible to speculate that different chemical characteristics, and consequently a modified interaction with the living vaginal epithelium, will also have an influence on the biological efficacy of the two MDs.

Both formulations can be used as a moisturizer and lubricant irrespective of the cause of vulvovaginal dryness, as well as a healing adjuvant by promoting the continued moisture of the vulvovaginal area for women suffering of discomfort and pain at vaginal mucosa level.

The biological system used to assess the MoA of the two MDs—hereafter called “Gel” and “Ovule”—has been a 3D reconstructed human vaginal epithelium (HVE) model in homeostatic physiological conditions and in modified culture conditions to recapitulate mucosal dryness.

Based on the protocol of Casiraghi et al. (2017) or existing internal methods, a HVE model has been used to reproduce the experimental conditions relevant to assessing the putative properties of the two MDs according to the claims associated with the following mechanisms:

- Film forming and persistency properties by caffeine permeation assay and Lucifer Yellow (LY) localization on HVE vertical sections to reach conclusions about their capacity to form a physical barrier on the vaginal epithelium.
- Re-epithelization and healing capacity on injured HVE tissues by trans-electrical epithelial resistance (TEER) measurements associated with histomorphological analysis (H&E) to reach conclusions about their capacity to restore epithelial barrier functionality.
- Moisturizing efficacy on HVE cultured in dry conditions by histomorphological analysis (H&E) and AQP3 expression and localization by IHC to evaluate their efficacy as lubricants and moisturizers. In parallel with the same protocol, it has been also possible to provide experimental evidence of the non-pharmacological action by investigating CD44 (hyaluronic acid receptor) expression and localization by IHC staining on “dry” HVE.

The parameters applied to the investigations have been selected to allow measurement of physical (TEER) chemical (caffeine passage) or morphological (H&E, HIC) modifications related to MD efficacy.

The originality of this study is the face-to-face evaluation of two HA-based SBMDs using approved and validated experimental protocols. The new *in vitro* data generated as well as observations already obtained in women generate some clinical perspectives for the use of the two Mucogyne^®^ SBMDs which are discussed at the end of this study.

## 2 Materials and methods

### 2.1 Test items

Mucogyne^®^ Gel and Mucogyne^®^ Ovule, both Class IIB according to MDR, were provided by Biocodex (France). The quali-quantitative formulation is reported in Table 1.

**TABLE 1:** Quali-quantitative formulations of Mucogyne^®^ Gel and Ovule.

Raw material	Mucogyne^®^ GEL (%) weight	Mucogyne^®^ OVULE (%) weight	Function
Purified water	88.58	—	Solvent
Propylene glycol	7	—	Solvent
Hydrogenated lecithin	1	—	Filming agent
Carbomer	0.75	—	Filming agent
Ethylhexylglycerin tocopherol	0.3	—	Antioxidant for excipients
Sodium hydroxyde	0.15	—	pH adjustment
Hexamidine diisethionate	0.1	—	Preservative
Sodium hyaluronate	0.1	0.22	Filming agent
Tocopheril acetate	0.02	0.43	Antioxidant for excipients
Propylen glycol, water, *Malva sylvestris* (mallow), and leaf extract	1	—	Antioxidant for excipients
Propylen glycol, water, *Chamomilla recutita* (matricaria), and flower extract	1	—	Stabilizer
Galactooligosaccharides	—	4.35	Excipient
Hydrogenated cocoglycerides	—	19.57	Excipient
Stearyl heptanoate	—	3.95	Excipient
Stearyl caprylate	—	1.70	Excipient
Hydrogenated palm kernel glycerides	—	69.78	Excipient

### 2.2 Test system

The reconstituted human vaginal epithelium (HVE/S) of 0.5 cm^2^ was manufactured by Episkin SA (Lyon, France) in accordance with ISO 9001. The model reproduces vaginal epithelium morphology: a structured epithelium is formed after 5 days of air-lift culture of immortalized cell line (A431) in a chemically defined medium. According to internal procedures, it has been used either at day 6 (mean tissue thickness is approximately 40–60 μm) or day 12 when the mean tissue thickness is approximately 130–170 μm. Before use, HVE batches were tested for the absence of hepatitis B, hepatitis C, *Mycoplasma* and HIV and have passed quality control based on histological analysis (well-stratified and non-keratinized squamous epithelium and number of cell layers). All this information is reported in the HVE shipment data sheet.

Upon reception in the laboratory, the HVE tissues were removed from the agarose nutrient solution under a sterile airflow cabin. The inserts were rapidly transferred to 6-well plates previously filled with maintenance medium (1 mL/well) (Episkin SA, Lyon, France) and incubated at 37°C, 5% CO_2_ and saturated humidity. No further controls were performed on the batch before use.

### 2.3 Materials and methods for film-forming and persistency model

Film-forming properties can be described as a temporary modification of permeation through the epithelium due to the product acting as a protective physical barrier (“film”) to caffeine passage across the tissue. The rationale for the film-forming protocol is based on a quantitative approach that uses caffeine [(MW = 194.2, ogP o/w = - 0.08 Merck Life Science srl, cat. C0750) low lipophilicity] with a well-known penetration kinetic as probe of barrier permeability (Casiraghi et al., 2017). The film-forming protocol also includes a series where the product is removed by gentle washing: this modification allows measurement of the film persistency as film-forming residual capacity mirroring the vaginal environment in the presence of physiological fluids. The two protocols were performed on a biological triplicate.

#### 2.3.1 HVE treatment

The culture inserts were placed in 6-well plates previously filled with 1 mL/well saline solution (basolateral compartment, receptor fluid). A positive control (PC) of 50 µL of Gel, Ovule, and white Vaseline (Laboratori Chimici Sella srl, Schio Italy) was applied directly onto the vaginal epithelium surface for 1 h at 37°C, 5% CO_2_, saturated humidity. Negative control (NC) tissues were untreated.

After 1 h treatment, 100 μL of 0.5% w/v caffeine (Merck Life Science srl) solution in ultrapure water was applied for 3 h to the apical compartment on each HVE without product removal for the film-forming protocol (PROTOCOL A). For the persistency protocol, the products and the PC were gently washed off after 1 h treatment with 0.5 mL of saline solution (NaCl 0.9%, EUROSPITAL) three times before caffeine application (PROTOCOL B). Caffeine penetration was monitored by collecting the receptor fluids (1 mL) from the basolateral compartment at 1 h and 3 h of exposure to caffeine solution.

#### 2.3.2 Analytical method for caffeine quantification

Receptor fluid samples were stored at 4°C before analysis of their caffeine concentration by UPLC-UV method using the 1290 Infinity II LC System (AGILENT Santa Clara, CA, United State) equipped with a C18 reversed-phase column (ACQUITY UPLCBEH-C18, 1.7 μm, 100 mm × 2.1 mm, WATERS CORPORATION, MA, United State) set at 25°C. A 5 μL sample was injected for isocratic elution at 0.25 mL/min. The composition of the eluent was 80% water/20% methanol. The wavelength was set at 273 nm.

For the UPLC method validation, standard linear calibration curves for caffeine (0.1 and 25 mg/L) were used (linearity *R* ^2^ ≥ 0.99, recovery %: 95%–105%). A total of 0.9% NaCl solution was used as medium for the standard curve preparation. The run time for caffeine elution was 3.5 min (LOQ ≤ 0.10 μg/mL, LOD = 0.017 μg/mL).

The results are expressed in micrograms and percentage of diffused caffeine compared to the applied dose as well as in comparison to the negative control (see Equation 1 below).

$$Caffeine\;\%=\frac{µg\;\text{Caffeine}\;\text{applied}}{µg\;\text{Caffeine}\;\text{found}}\;x\;100.$$ (1)

### 2.4 Material and method for epithelial permeability by Lucifer Yellow (LY) localization on histological sections

HVE tissues were exposed to the MDs according to the protocols for film forming and persistency described in Section 2.3.1: total 4 h treatment for film forming and 1 h +3 h after washing for persistency. In both protocols, the NC was untreated. We applied 0.5 mL of Lucifer Yellow (500 μM in saline solution, Merck Life Science) to the apical compartment (into the insert) of the tissue. Saline solution (1 mL) was added into the basolateral compartment, and LY was left to incubate for 1 h at 37°C. At the end of the treatment, the tissues were fixed in formalin solution neutral buffered, 10%. After fixation, the HVEs in triplicates were included in the same paraffin block, obtaining 5 μm sections. Tissue sections were rehydrated and mounted using FluoroshieldTM with DAPI (4′,6-diamidino-2-phenylindole) nuclear counterstaining.

The histological sections were visualized with Microscope THUNDER imager 3D, acquired with camera K5 (fluorescence), and processed with LASX 3.7.5 software. For each experimental condition, three biological replicates were assessed. For each biological replicate, two slides were analyzed.

### 2.5 Materials and method for re-epithelization and moisturizing properties on lesional HVE

Re-epithelization efficacy was evaluated on lesional HVE; the epithelial barrier was impaired, creating tissue discontinuity by a reproducible procedure consisting of four cross injuries (#), performed using a glass capillary tip, 1 h before starting the treatment with the product. The injury induces a barrier impairment and modifies the epithelial architecture mirroring a superficial wound: this series represents the untreated injured control (INJ). The negative control (NC) tissues were non-lesioned and untreated. In the treated series, 50 μL or 50 mg of product were homogeneously applied on the HVE surface for 24 h exposure at 37°C, 5% CO_2_, saturated humidity.

At the end of the exposure, the residual product was gently washed out with Dulbecco’s Phosphate Buffered Saline (DPBS) (Merck Life Science), and the tissues were collected for:

• TEER measurement to analyze the recovery of the superficial lesions and to reach conclusions about barrier function integrity (Section 2.8).
• H&E staining to investigate the recovery of the superficial lesions and re-epithelizing properties (Section 2.9).

The analysis was performed on biological triplicate.

### 2.6 Materials and method for the dryness model

The dryness condition was induced by incubating HVE tissues in modified environmental conditions (R.H. ≤ 40%, T° = 40°C and 5% CO_2_) for 48 h. The products were then applied on the “DRY” HVE model for 4 h in standard culture conditions (37°C, 90% R.H. and 5% CO_2_). HVE tissues cultivated in these conditions mirror the discomfort associated with dryness and water balance impairment. The protocol was performed on a biological triplicate. After 4 h treatment, the tissues were collected for:

• H&E staining to investigate morphological modifications;
• AQP3 protein expression and localization as response to dryness stress by immunostaining;
• CD44 (glycoprotein, hyaluronic acid receptor) expression by immunostaining.

Investigating AQP3 expression in the suprabasal layer of the vaginal epithelium evaluates the capacity of MDs to stimulate an increase of water content as lubricant and to counteract dryness.

Investigation of CD 44 was used to confirm the MDs’ principal mechanism of action by physical-mechanical means.

### 2.7 Trans-epithelial electrical resistance (TEER) measurement

TEER is an indirect assessment of the stability of tight junctions and is used to measure barrier function in epithelial tissues. It reflects the global resistance of the barrier linked both with the tissue’s structure and its thickness.

TEER was measured using a Millicell®-ERS (Electrical Resistance System) instrument (Millipore Corporation, Bedford, MA, United State). The measure was performed on all HVE series at the end of the expos; tissues were placed in 6-well plates previously filled with 5 mL/well saline solution, and 0.5 mL of saline solution were applied on the tissue’s surface. The Millicell-ERS instrument was placed with the electrodes in the two chambers, and the TEER was measured (range 0–20 kΩ). Three measurements for each tissue were made, and the mean TEER value of each tissue was calculated. The mean TEER value was then corrected considering the tissue surface (0.5 cm^2^) according to the following formula (see Equation 2 below):

$$\Omega /{\text{cm}}^{2}=\text{tissue}’s\text{ mean TEER }\left(\Omega \right)\;x\text{ tissue surface }\left(0.5{\text{cm}}^{2}\right).$$ (2)

The mean TEER values of each series were then calculated and expressed as Ω/cm^2^.

### 2.8 Hematoxylin and eosin (H&E) staining

At the end of the treatment, tissues (intact, injured, or “dry” according to the protocol) were fixed in formalin solution, and neutral buffered 10% (Merk Life Science). According to the internal procedure (LAB-M-016), the samples were then included in paraffin (Merk Life Science) blocks, obtaining sections of 5 μm. Slides were stained with hematoxylin and eosin following internal procedures; hematoxylin stains cells blue whereas eosin stains the extracellular matrix and cytoplasm pink. The histological samples were analyzed in triplicate under light microscopy using LEICA DMi8 THUNDER imager 3D composed by camera DFC 450 C and LASX 3.7.5 software (×20 magnification).

### 2.9 AQP3 and CD44 detection by immunofluorescence on stressed HVE models

In order to monitor the dynamic of the moisturizing properties, two relevant biomarkers—water channel modification (AQP3) and hyaluronic acid (CD44)—were investigated. Immunostaining of AQP3 and CD44 on HVE cultured in dry conditions was performed using Anti-AQP3 (Abcam, # Ab153694) and Anti-CD44 (EuroClone, 156-3C11) as primary antibodies. The two IHC methods are reported in the internal procedures (LAB- M-022), with the measurement times in the dryness model conditions adapted from (Reference VitroScreen).

The signal was visualized with HISTOFINE SIMPLE STAIN AP MULTI alkaline phosphatase (NICHIREI BIOSCIENCES, 414262F) and new fuchsin substrate kit as chromogen (NICHIREI BIOSCIENCES, 415161F) for AQP3; nuclei were counter-stained with GILL III hematoxylin. The CD44 detection was visualized with Alexa Fluor Plus 488 goat anti-mouse (Life Technologies, A32723); nuclei were counter-stained with DAPI solution.

The stained samples were analyzed in triplicate under light microscopy using LEICA DMi8 THUNDER imager 3D composed by camera K5 and LASX 3.7.5 software. For each biological replicate, the entire tissue sections were acquired at ×20 magnification by Leica LASX Tilescan technology both for lesioned and unlesioned series, and AQP3 and CD44 signals were quantified on them; the software highlights the signal and quantifies it using the sum intensity as parameter. Statistical analysis was performed using one-way ANOVA followed by *post hoc* two-per-two comparisons with negative control using Tukey’s test when a significant effect was detected.

### 2.10 Statistical analysis

All measurements were performed on triplicate HVEs tissues. One-way ANOVA followed by a *post hoc* Tukey HSD test (*p* < 0.005) was performed using GraphPad Prism version 9.2.0 (GraphPad Software, San Diego, CA, United State). The products and the white Vaseline were compared with the untreated negative control, and statistically significant results were reported as *p*-values.

## 3 Results

### 3.1 Film forming and persistency properties on HVE

#### 3.1.1 Film forming

The effects of Mucogyne Gel and Ovule are reported in Table 2 expressed as the percentage of caffeine found in the basolateral compartment compared to the applied dose (0.5 mg). In Table 3, the percentage of caffeine passage reduction is reported as normalized with respect to the reduction of caffeine passage in the negative control.

**TABLE 2:** % of caffeine quantified in the receptor fluid at 1 h and 3 h of caffeine exposure (PROTOCOL A).

Product	Caffeine % in basolateral compartment, compared to dose applied (PROTOCOL A)	
	1 h	3 h
Negative control (NC)	13.5 ± 0.6	27.7 ± 0.8
White Vaseline	1.7 ± 0.7***	6.1 ± 1.6***
Gel	9.1 ± 0.9***	22.0 ± 1.4**
Ovule	5.0 ± 0.9***	14.5 ± 2.1***

Values reported as mean ± SD (ANOVA test *post hoc* Tukey’s versus NC; **: *p* < 0.01, ***: *p* < 0.001).

**TABLE 3:** % of caffeine passage reduction (PROTOCOL A).

Product	Film-forming efficacy (PROTOCOL A)	
	Caffeine passage reduction vs. NC	
	1 h	3 h
White Vaseline	−87%***	−78%***
Gel	−33%***	−20%**
Ovule	−63%***	−48%***

Values reported as means (ANOVA test *post hoc* Tukey’s versus NC; **: *p* < 0.01, ***: *p* < 0.001).

As shown in Tables 2 and 3, the positive control—white Vaseline—confirms its film forming capacity by blocking caffeine passage (87% and 78% at 1 h and 3 h, respectively), validating the experimental system.

The Ovule showed the best film-forming capacity; after 1 h exposure, it determined a statistically significant reduction of caffeine passage of 63% (*p* < 0.001), and the film-forming capacity was still significant (48%) after 3 h (*p* < 0.001).

The Gel determined a statistically significant reduction of caffeine passage: 33% (*p* < 0.001) at 1 h and 20% after 3 h (*p* < 0.01).

These data underline that both products have film-forming properties. According to their different physical characteristics, the Ovule (lipophilic formulation) shows a higher capacity to form a protective barrier (film-forming efficacy) on the vaginal epithelium in the adopted experimental conditions.

#### 3.1.2 Film persistency

The results for the film persistency (i.e., residual film-forming activity following a gentle wash) protocol for Gel and Ovule are reported in Table 4. Results are expressed as the percentage of caffeine found in the basolateral compartment compared to the applied dose (0.5 mg). In Table 5, the percentage of caffeine passage reduction is reported as normalized to the reduction of caffeine passage in the negative control.

**TABLE 4:** % of caffeine quantified in the receptor fluid at 1 h and 3 h of caffeine exposure (persistency, PROTOCOL B).

Product	Caffeine % in basolateral compartment compared to dose applied	
	1 h	3 h
Negative control (NC)	12.6 ± 0.7	27.8 ± 0.8
White Vaseline	1.3 ± 0.1***	5.5 ± 0.7***
Gel	12.2 ± 1.1^ns^	26.0 ± 2.3^ns^
Ovule	3.5 ± 0.2***	11.7 ± 0.4***

Values reported as mean ± SD (ANOVA test *post hoc* Tukey’s versus NC; ***: *p* < 0.001; ns, not significant).

**TABLE 5:** % of caffeine passage reduction (persistency, PROTOCOL B).

Product	Film persistency efficacy (PROTOCOL B)	
	Caffeine passage reduction vs. NC	
	1 h	3 h
*White Vaseline*	−90%***	−80%***
*Gel*	−3%^ns^	−7%^ns^
*Ovule*	−73%***	−58%***

Values reported as means (ANOVA test *post hoc* Tukey’s versus NC; ***p < 0. 001; ns: not significant).

In the HVEs treated with white Vaseline, the caffeine quantified in the basolateral compartment corresponds to 1.3% and 5.5% (respectively after 1 h and 3 h) of the applied dose, confirming its film forming capacity also after a washing procedure corresponding to 90% at 1 h and 80% at 3 h compared to the NC.

The Gel applied during 1 h followed by washing was not effective in terms of the persistency of the film formed on the HVE surface, and a non-significant reduction of caffeine passage at both timepoints was quantified (Table 5).

On the other hand, the Ovule induced a significant caffeine passage reduction: 73% (*p* < 0.001) at 1 h and 58% after 3 h (*p* < 0.001), confirming the influence of the chemical-physical characteristics of the formulation on the expected biological effects.

#### 3.1.3 Lucifer Yellow localization

**FIGURE 1:**
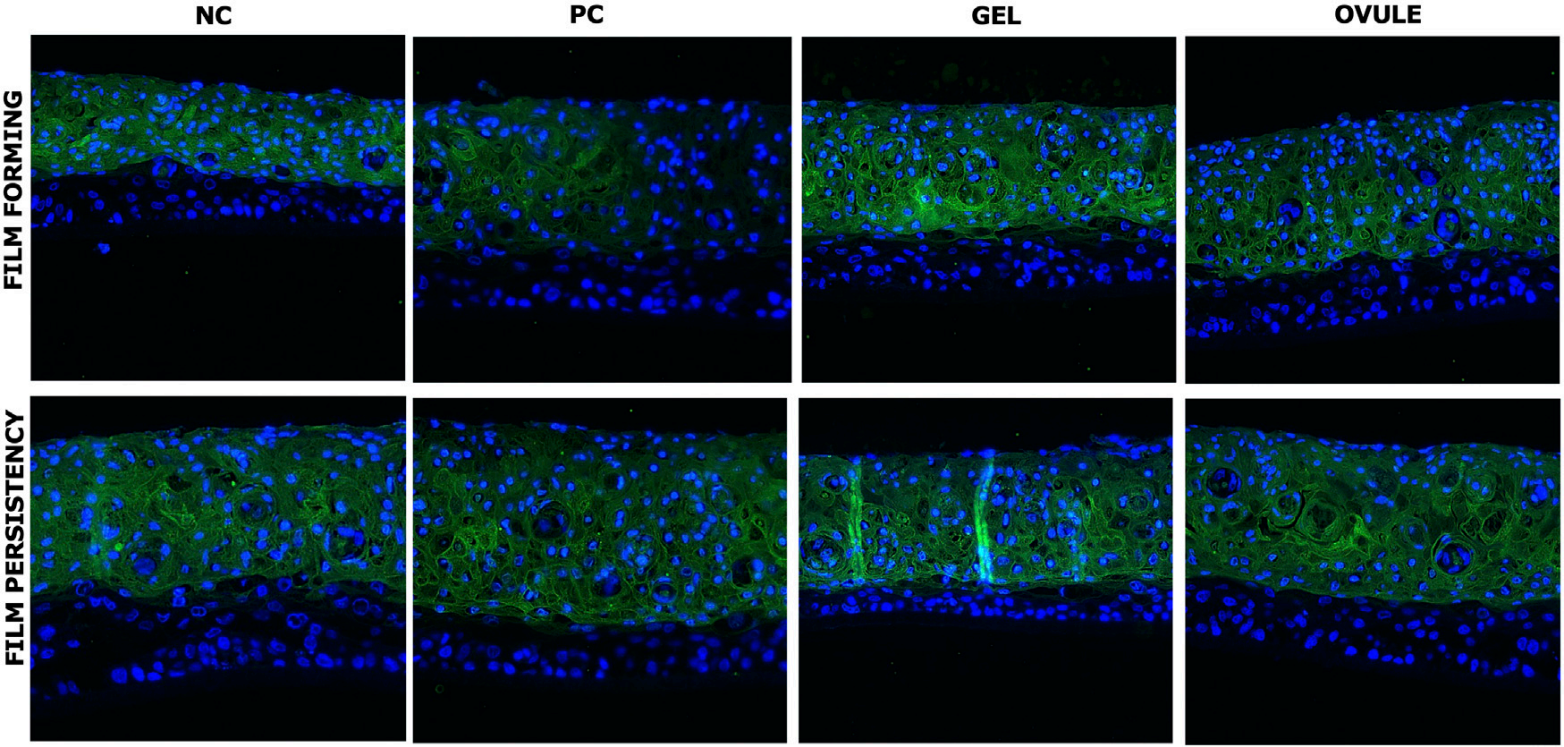
LY localization after exposure to products (GEL or OVULE) or PC (white Vaseline) or untreated tissues (NC) according to film-forming and film persistency protocols. LY in green; nuclei in blue. ×20 magnification.

In Figure 1, the localization of LY probe on HVE vertical histological sections is presented for the film-forming and film persistency protocols.

On the untreated series (NC), as expected, LY diffused through the superficial squamous layer, characterized by reduced cell–cell adhesion. Its penetration into the deeper epithelium was blocked by the integrity of intermediate layer, whose function is to increase the tensile strength of the epithelium and ensure its compactness.

In the HVEs treated with white Vaseline, a specific fluorescence distribution was observed, characterized by a limited and non-homogenous penetration of LY. The well-known occlusive properties of the white Vaseline are probably responsible for these results, which globally confirm the results found on caffeine quantification.

In both series of HVEs treated with Gel and Ovule, LY penetrates into the more superficial squamous layer, as observed in the untreated series, and it remained fully blocked by the intermediate layer structure in both protocols. This suggests a physiological interaction of both MDs with the upper layers of vaginal epithelium structure, confirming the film-forming properties observed in the caffeine assay and without exerting an occlusive action or interfering with the epithelium’s physiology.

### 3.2 Re-epithelization on lesional HVEs

#### 3.2.1 TEER measurements

**FIGURE 2:**
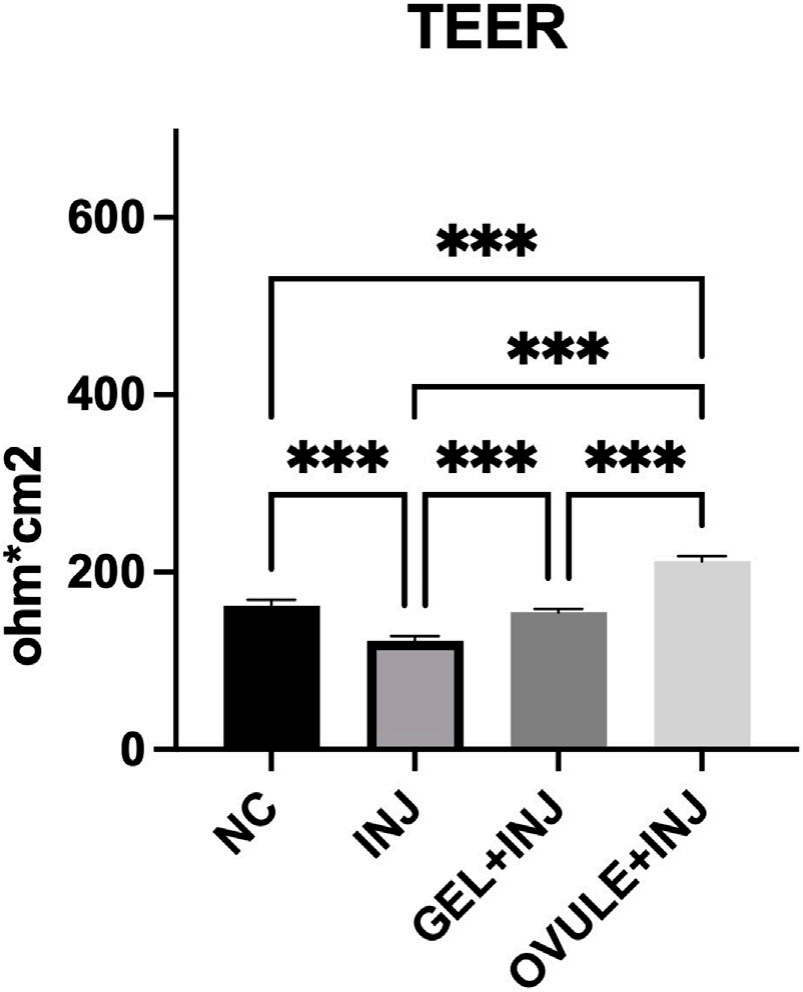
TEER expressed in ohm*cm^2^ (values reported as mean ± SD) of HVE after 24 h from injury. Negative control (NC): not lesioned and untreated HVE cultured for 24 h in standard culture conditions (37°C 5% CO_2_). Injured HVE (INJ): HVE after 24 h treatment with Gel (GEL + INJ) and Ovule (OVULE + INJ). The value reported is the average of three measurements performed on the same tissue. Statistical analysis: ANOVA; ***: statistical analysis performed by one-way ANOVA *post hoc* Tukey; ***p < 0.001.

In Figure 2, the results of TEER measured on HVEs series and expressed in ohm*cm^2^ are reported.

As expected, the injury procedure caused a significant higher decrease in TEER values (122.6 Ω*cm^2^) than in untreated non-lesional control (162 Ω*cm^2^).

A significant efficacy of the two MDs in restoring barrier function and integrity has been shown: in particular, TEER values are significantly increased in lesional HVEs following 24 h treatment with Gel (154.5 Ω*cm^2^) and Ovule (212 Ω*cm^2^), corresponding to an increase in TEER values of 26% and 73% for the Gel and the Ovule, respectively, compared to the injured control.

The TEER measure depends on the integrity of the TJ’s structure in the epithelium and on the thickness of the tissue; it is thus possible to conclude that the TEER increase reflects a higher functionality of the epithelial barrier. These data confirm, on one hand, the film-forming properties and physiological interaction on the MDs with the upper layer of the epithelium and, on the other hand, the capacity to restore barrier function integrity in injured HVE.

#### 3.2.2 H&E staining

**FIGURE 3:**
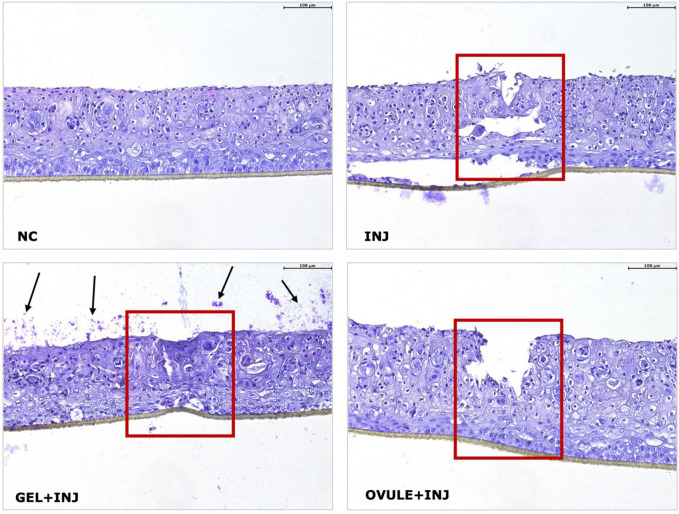
H&E staining of lesional HVE after 24 h treatment with Gel (GEL + INJ) and Ovule (OVULE + INJ). Negative control (NC): not lesioned and untreated HVE cultured for 24 h in standard culture conditions (37°C 5% CO_2_). Injured HVE (INJ): lesional HVE untreated and cultured for 24 h in standard conditions. Black arrows indicate residual non-penetrated gel formulation visible on the epithelial surface (squamous layer) along the whole section surface. The red box indicates the lesional area. ×20 magnification.

Figure 3 presents the histo-morphological images of HVE lesional tissues, thereby allowing a comparison with the non-lesional negative control (NC).

In the NC, a regular morphology of HVE at day 12 is observed, consisting of a non-keratinized differentiated superficial squamous layer with weak cell–cell contacts.

Basal layer reference morphology is perfectly observed. In the injured HVE tissue (INJ), the epithelial continuity is broken, and cellular damage is observed inside and around the lesioned area, consisting of many pyknotic nuclei; morphological modifications suggesting a reduced tissue integrity are also observed in the squamous and basal layers.

After 24 h treatment with the Gel, residual amounts of non-penetrated gel formulation are visible on the epithelial surface (squamous layer) along the whole section surface, confirming its film forming properties (Figure 3; black arrows in GEL + INJ). Compared to the lesioned control, the epithelial discontinuity induced by the injury appears partially restored, and the squamous epithelium integrity appears better preserved by the Gel than the injured control.

Treatment with the Ovule not reduced the epithelial discontinuity, but globally, the morphology appears to be better preserved throughout the tissue section, with a reduction of pyknotic nuclei compared to the injured control.

### 3.3 Mechanism of action on dryness model: H&E staining and AQP3 localization and expression

**FIGURE 4:**
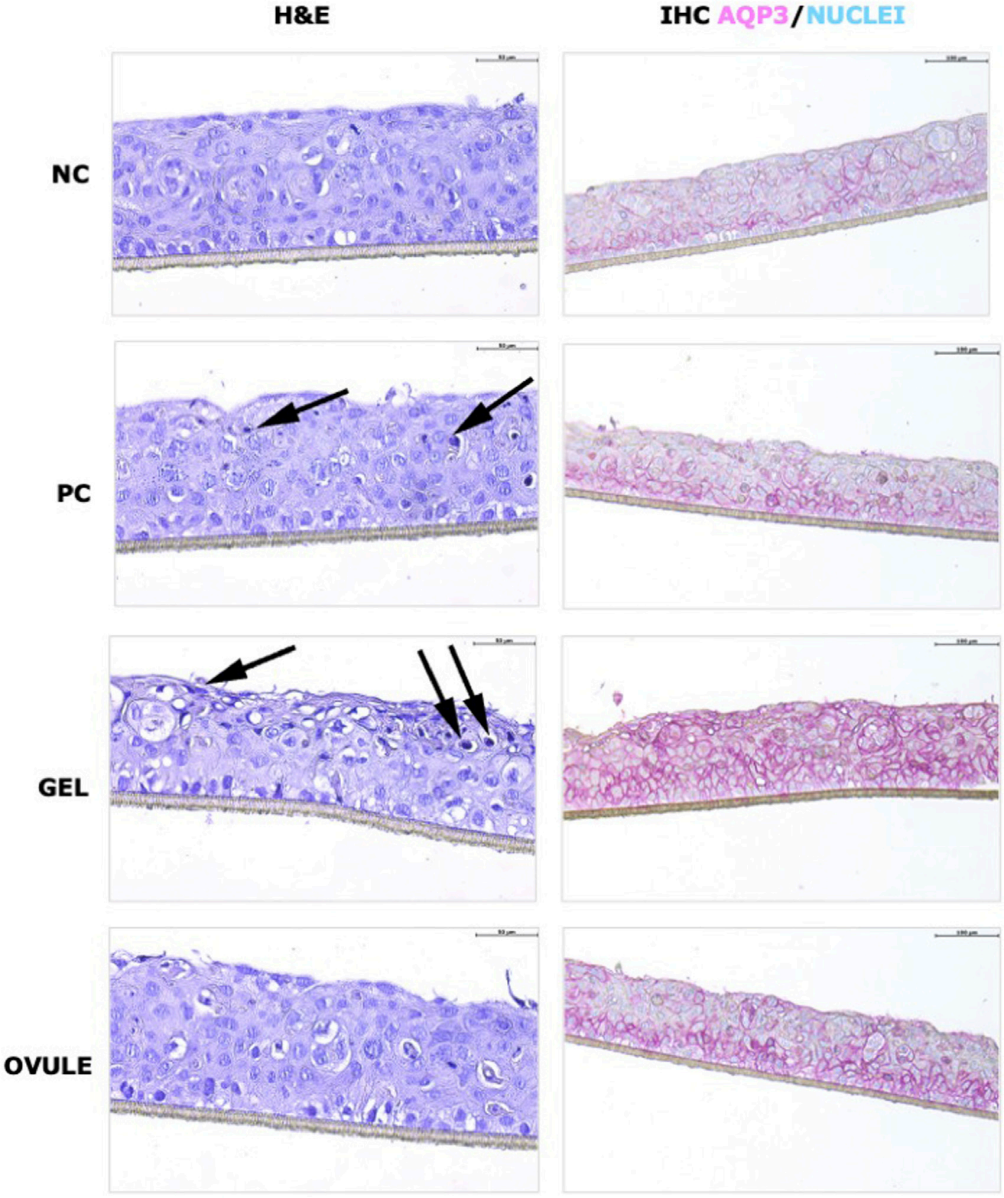
H&E staining (left) and AQP3 localization (right) on “dry” HVE tissues after 4 h treatment with Gel and Ovule. Normal HVE tissues treated with saline solution (NC) or dry HVE cultivated without any treatment (PC) or with Gel or Ovule for 4 h in standard conditions. Black arrows indicate pyknotic nuclei. ×20 magnification.

**FIGURE 5:**
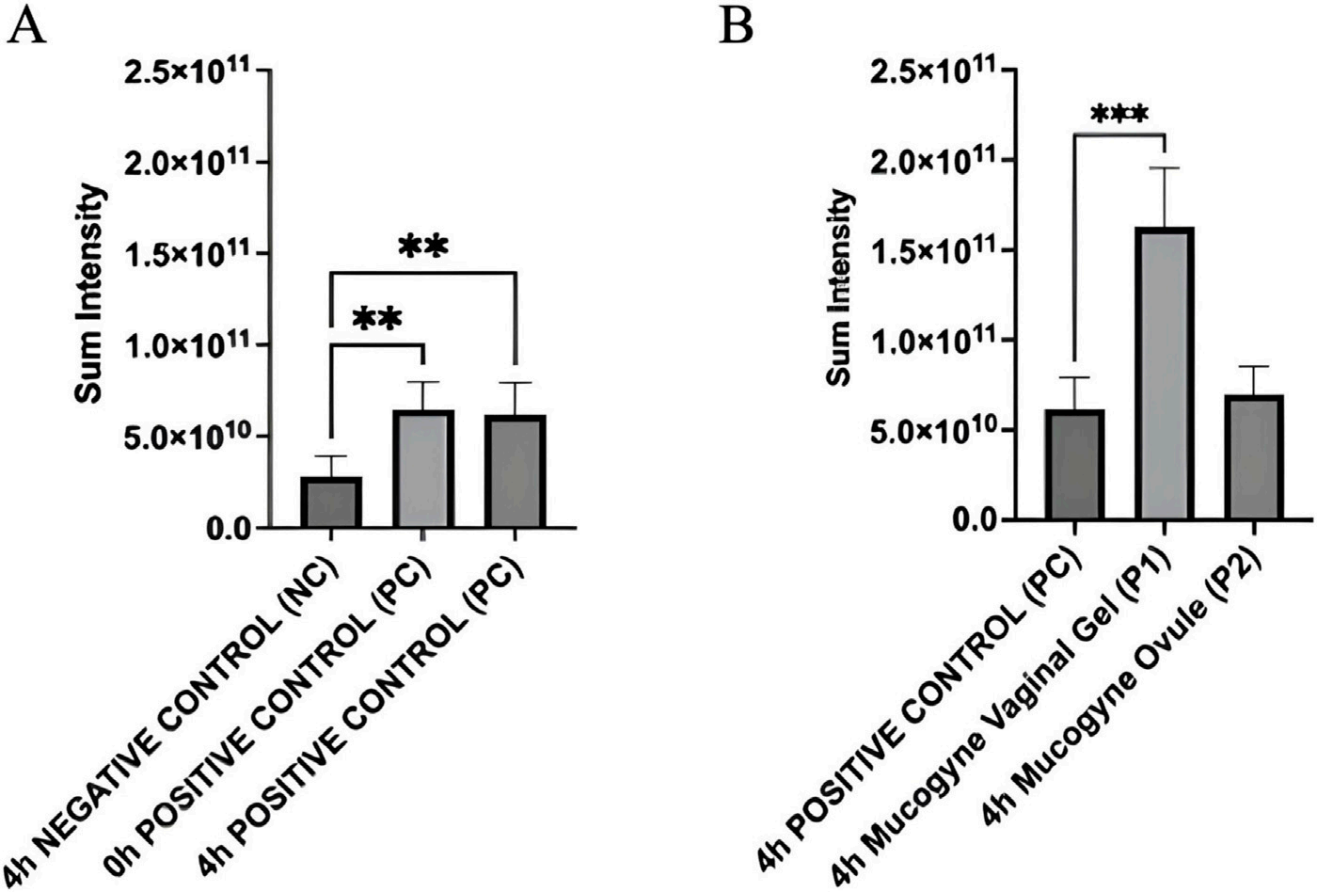
**(A)** Quantification of AQP3 expression in “dry” HVE tissues immediately, and 4 h after the end of the dryness induction (PC 0 h and 4 h). “Dry” HVE tissues treated with saline solution (NC) or cultivated without any treatment (PC) for 4 h in standard conditions. Intensity values are mean from three biological replicates. **(B)** Quantification of AQP3 expression in “dry” HVE treated tissues (P1 and P2) over 4 h compared to positive control (PC 4 h). Statistical analysis performed by one-way ANOVA and Tukey *post hoc* test: **p < 0.002 and ***p < 0.001.

Figure 4 presents H&E and AQP3 images by immunohistochemistry of “dry” HVE; included are the non-stressed negative control (NC) and stressed non-treated positive control (PC). In Figure 5, AQP3 protein expression is quantified.

The H&E results (Figure 4) suggest that in the positive control, as expected, the dryness stress has induced a morphological modification characterized by an increase of pyknotic cells in the intermediate layers visible after 4 h from dryness induction.

As shown in Figure 5A, the AQP3 was quantified as significantly higher after dryness stress, and the expression was stable within 0 h and 4 h within the basal layer (Figure 4). Dryness stress determined a defense response, increasing AQP3 expression in the deeper tissue layers to significantly recruit more water from the basolateral compartment.

The morphology of HVEs cultivated in dryness and then treated with the Gel show the strong affinity of the hydrophilic gel formulation to the dry epithelium, suggesting that a more permeable epithelium has quickly absorbed the hydrophilic gel to restore the water physiological content: some pyknotic nuclei were detected in the zone, showing a strong interaction of the Gel with the squamous layer (Figure 4).

A statistically significant increase of AQP3 protein was measured for the HVEs treated with the Gel (*p* < 0.001) (Figure 5B): a strong signal is visible in intermediate and superficial layers; these results agree with those for H&E, suggesting that AQP3 water channels were activated in the dry superficial layer moisturized by the hydrophilic Gel and indicating a direct activity on water channels of hydrophilic ingredients by a chemical mechanism of action. The correlation between AQP3 increase and topical treatment with hydrophilic active ingredients is well-known in case of barrier impairment and dryness (Garcia et al., 2011; Bollag et al., 2020).

In the HVEs treated with Ovule (4 h P2), no morphological differences were detected in comparison to the dry control (4 h PC), suggesting a different interaction with the squamous layer characterized by a predominant film forming with superficial distribution of the formulation on the “dry” HVE (Figure 4). This was further confirmed by AQP3 expression (Figure 5B), which was not significantly different in term of signal intensity from the dryness control (4 h PC). However, the Ovule localization within the HVE was found to be different, with a principal distribution in the basal layer that suggested an efficient moisturizing mechanism by reducing water evaporation thanks to its predominant film forming properties.

#### 3.3.1 CD44 localization and expression

**FIGURE 6:**
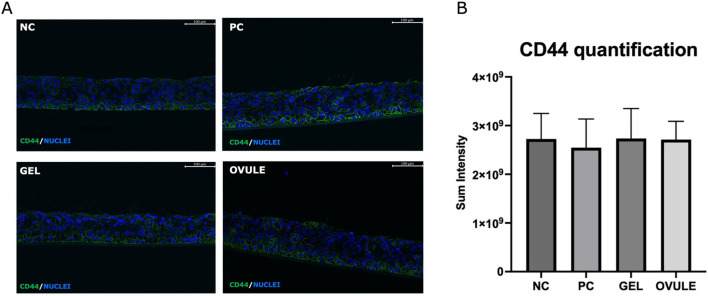
CD44 localization **(A)** and quantification **(B)** by immunofluorescence in “dry” HVE tissues after 4 h treatment with Gel and Ovule. “Dry” HVE tissues treated with saline solution (NC) or cultivated without any treatment (PC) for 4 h in standard conditions. Intensity values are means from three biological replicates. Statistical analysis performed by one-way ANOVA: no significant differences calculated.

In Figure 6, immunofluorescent staining of CD44 in “dry” HVE is presented (6A) and quantified (6B).

HA is a major component of tissue matrices and fluids and is involved in a variety of physiological functions such as maintaining tissue hydration and osmotic balance, cell proliferation, adhesion, and migration. CD44 is a glycoprotein that is widely expressed on the surface of many mammalian cells, and it is the selective receptor for hyaluronic acid.

No significant modification of the localization (Figure 6A) and expression of CD44 receptor (Figure 6B) was revealed in the PC, indicating that dryness stress did not involve the HA receptor in the adopted experimental conditions (after 4 h recovery time).

No significant modification in CD44 localization and expression compared to the NC was detected in samples treated with Gel and Ovule: both formulations based on different forms of hyaluronic acid showed a moistening property without involving the HA receptor, in accordance with their non-pharmacological mechanism of action in the adopted experimental window.

## 4 Discussion

In the project described in this work, a non-clinical, ethical approach based on the use of 3D reconstructed vaginal epithelium has been adopted to provide scientific evidence on their mechanisms of action according to MDR requirements on MDs for vaginal use.

A summary of the experimental models applied to these investigations is reported in Table 6.

**TABLE 6:** Biological *in vitro* models, claims, and endpoints assessed to investigate the mechanisms of action of mucogyne gel and mucogyne ovule intended for vaginal use.

Mechanism type	Biological model	Experimental PROTOCOL	Testing parameters
Mechanical	HVE at day 12	Film-forming film persistency after washing procedure	Caffeine permeation through the epithelium without product removal
			Caffeine permeation through the epithelium after product removal
			LY diffusion in histological vertical sections
Physical	Injured HVE at day 12	Re-epithelization	TEER
			H&E
Chemico-physical	HVE at day 5 cultured in dryness conditions	Dryness	H&E
			AQP3 localization and expression by IHC staining
Non-pharmacological activity		HA receptor activation	CD44 expression by IF staining

HVE, human vagina epithelium; LY, Lucifer Yellow; TEER, trans-electrical epithelial resistance; H&E, hematoxylin and eosin; AQP3, aquaporin 3; IHC, immunohistochemistry; CD44, glycosylate protein receptor of hyaluronic acid; IF, immunofluorescence.

Based on caffeine passage results, it is possible to conclude a good film forming property for the Gel applied on the vaginal epithelium with a statistically significant reduction of caffeine passage of 33% and 20% after 1 h and 3 h, respectively. The same behavior is observed for the Ovule, for which a statistically significant reduction of caffeine passage of 63% and 48% after 1 h and 3 h, respectively, was found.

The film persistency capacity of Mucogyne Ovule was superior to Gel: Ovule has significantly reduced caffeine passage of 73% and 58% at 1 h and 3 h, respectively, supporting the long-lasting effects of Ovule on the vaginal epithelium. The lipidic nature of the Ovule provides long-lasting effectiveness by allowing the controlled release of hyaluronic acid through the vaginal mucosa.

The results of LY localization by fluorescence evaluation on histological HVEs sections confirmed that the barrier functionality and integrity were fully preserved with both products (with and without product washing) during the study.

In the presence of a lesion, the significant increase of TEER values after 24 h treatment with Gel (+26%) and Ovule (+73%) compared to the injured control confirms the film-forming properties of the products, with Ovule delivering a superior protection thanks to these properties.

The histo-morphological analysis on lesioned tissues supports the presence of Gel residual on the epithelium surface after 24 h of treatment and confirms its re-epithelization properties.

In the dryness model, the data support a specific affinity of the hydrophilic Gel formulation to the “dry” HVE, determining a direct moisturizing effect confirmed by the HIC results on AQP3 (localization and expression). On the contrary, a superficial distribution of the Ovule formulation on the “dry” HVE supported by H&E staining images and AQP3 expression and localization suggests an indirect moisturizing mechanism by reducing water evaporation thanks to its predominant film-forming properties. The AQP3 role in tissue hydration has been reported in the literature (Hara and Verkman, 2003; Hara-Chikuma and Verkman, 2008; Park et al., 2008; Kim et al., 2009; Kim et al., 2011; Guo et al., 2013; Verkman et al., 2014; Kunovac Kallak, 2015).

The results of CD44 localization and expression after treatment with the products did not differ from NC (HVE cultured in standard conditions) and positive control (non-treated “dry” HVE), suggesting that they did not interact with the HA receptor and confirming a mechanical chemical-physical principal mechanism of action.

The present *in vitro* experimental approach on 3D human vaginal epithelium proved suitable for investigating the mechanisms of action of the two MDs formulations. Experimental data produced in the different efficacy models have been analyzed for their statistical significance and robustness in order to draw conclusions about the relevance of the principal claims associated with the two MDs: maintaining a physiological natural moisture of the vulvovaginal mucosa to protect the epithelial barrier in case of functional impairment, and lubricating the mucosa acting with the chemical-physical mechanism of action thanks to their physic-chemical properties. Furthermore, the non-clinical *in vitro* approach described here provides evidence-based data and supports the value of non-animal testing methods that can be performed that mirror the products efficacy with doses closer to ones in use and on their specific site of action.

Among the clinical perspectives concerning Mucogyne^®^ SBMDs, one of the main indications would be postmenopausal vaginal dryness. A decline in endogenous ovary function leads to atrophic changes of the vagina and other estrogen-dependent tissues. The quality of life of menopausal women can be negatively affected by vulvovaginal atrophic changes. More than half of postmenopausal women will have urogenital discomfort associated with estrogen deficiency (Iosif and Bekassy, 1984; Bachmann, 1995; Notelovitz, 1997), especially dryness, soreness, irritation, discharge, and dyspareunia.

Such symptoms were long neglected or even ignored. Today’s menopausal women are more sexually active (Thornton et al., 2015), less accepting of such symptoms, and seek a better quality of life (Ling and Wang, 2023). Oral hormone replacement therapy (HRT) can reduce climacteric symptoms, but sometimes only imperfectly. Furthermore, HRT has been consequently reconsidered due to adverse effects (Santoro et al., 2021). In symptomatic women who have no other indications for systemic hormone replacement or prefer not to use systemic therapy, local vaginal treatment with estrogen is also an effective option in preventing atrophic vaginal changes and relieving symptoms (Notelovitz, 1997; Messinger-Rapport and Thacker, 2001). Because the vagina loses water-retaining ability at menopause, hyaluronic acid can be another option to repair processes of the atrophic and dystrophic states of the vagina and age-related dryness due to estrogen deficiency. Past studies have shown that hyaluronic acid vaginal products could significantly improve epithelial atrophy and provide relief for vaginal symptoms in a manner comparable to local estrogenic treatment (Ekin et al., 2011; Chen et al., 2013). Thus, HA products can be used in patients with atrophic vaginitis as an alternative for women who do not want to use hormones or in addition to hormonal treatments.

The use of HA could also be considered for perineal surgery or after perineal trauma (episiotomy, perineal tears) during the puerperium. Perineal trauma is a common complication of childbirth in developed countries. According to the 2021 national perinatal survey, 8.3% of women in France will have an episiotomy and 58.8% a perineal tear (https://www.santepubliquefrance.fr/docs/enquete-nationale-perinatale.-rapport-2021.-les-naissances-le-suivi-a-deux-mois-et-les-etablissements). Perineal trauma can lead to healing disorders, perineal pain, or altered sexuality, especially dyspareunia (Glazener et al., 1995). Limited research has been directed to managing perineal trauma *postpartum*. To the best of our knowledge, the use of HA in this context has never been systematically evaluated. Nonetheless, there is potential value in exploring this application to promote and expedite healing, leading to reduced pain, enhanced comfort, and improved sexual wellbeing. The concept of applying HA in the form of a gel is particularly intriguing as it may help women reclaim a part of their bodies altered following perineal trauma and, more significantly, who have undergone a substantial physiological event such as vaginal childbirth. Nevertheless, further studies will be needed to validate these hypotheses.

## Data Availability

The raw data supporting the conclusions of this article will be made available by the authors, following reasonable request.

## References

[B1] Ayehunie S. Cannon C. LaRosa K. Pudney J. Anderson D. J. Klausner M. (2011). Development of an *in vitro* alternative assay method for vaginal irritation. Toxicology 279 (1–3), 130–138. 10.1016/j.tox.2010.10.001 20937349 PMC3003762

[B2] Ayehunie S. Wang Y. Y. Landry T. Bogojevic S. Cone R. A. (2018). Hyperosmolal vaginal lubricants markedly reduce epithelial barrier properties in a three-dimensional vaginal epithelium model. Toxicol. Rep. 5, 134–140. 10.1016/j.toxrep.2017.12.011 29854584 PMC5977164

[B3] Bachmann G. (1995). Urogenital ageing: an old problem newly recognized. Maturitas 22 (Suppl. l), S1–S5. 10.1016/0378-5122(95)00956-6 8775770

[B4] Benini V. Ruffolo A. Casiraghi A. Degliuomini R. Frigerio M. Braga A. (2022). New innovations for the treatment of vulvovaginal atrophy: an up-to-date review. Medicina 58 (6), 770. 10.3390/medicina58060770 35744033 PMC9230595

[B5] Bollag W. B. Aitkens L. White J. Hyndman K. A. (2020). Aquaporin-3 in the epidermis: more than skin deep. Am. J. Physiol. Cell Physiol. 318 (6), C1144–53. 10.1152/ajpcell.00075.2020 32267715 PMC7311736

[B6] Casiraghi A. Ranzini F. Musazzi U. M. Franzè S. Meloni M. Minghetti P. (2017). *In vitro* method to evaluate the barrier properties of medical devices for cutaneous use. Regul. Toxicol. Pharmacol. 90, 42–50. 10.1016/j.yrtph.2017.08.007 28822878

[B7] Ceriotti L. Buratti P. Corazziari E. S. Meloni M. (2022). Protective mechanisms of liquid formulations for gastro-oesophageal reflux disease in a human reconstructed oesophageal epithelium model. MDER 15, 143–152. 10.2147/MDER.S363616 PMC9124487 35610977

[B8] Chen J. Geng L. Song X. Li H. Giordan N. Liao Q. (2013). Evaluation of the efficacy and safety of hyaluronic acid vaginal gel to ease vaginal dryness: a multicenter, randomized, controlled, open-label, parallel-group, clinical trial. J. Sex. Med. 10 (6), 1575–1584. 10.1111/jsm.12125 23574713

[B9] Costa A. P. F. Sarmento A. C. A. Vieira-Baptista P. Eleutério J. Cobucci R. N. Gonçalves A. K. (2021). Hormonal approach for postmenopausal vulvovaginal atrophy. Front. Reprod. Health 3, 783247. 10.3389/frph.2021.783247 36303971 PMC9580661

[B10] Costin G. E. Raabe H. A. Priston R. Evans E. Curren R. D. (2011). Vaginal irritation models: the current status of available alternative and *in vitro* tests. Altern. Lab. Anim. 39 (4), 317–337. 10.1177/026119291103900403 21942546

[B11] De Jong W. H. Hoffmann S. Lee M. Kandárová H. Pellevoisin C. Haishima Y. (2018). Round robin study to evaluate the reconstructed human epidermis (RhE) model as an *in vitro* skin irritation test for detection of irritant activity in medical device extracts. Toxicol Vitro 50, 439–449. 10.1016/j.tiv.2018.01.001 29326048

[B12] De Servi B. Meloni M. Saaid A. Culig J. (2020). *In vitro* comparison of safety and efficacy of diluted isotonic seawater and electrodialyzed seawater for nasal hygiene. MDER 13, 391–398. 10.2147/MDER.S285593 PMC7726834 33312003

[B13] De Servi B. Ranzini F. Piqué N. (2016). Effect of utipro^®^ (containing gelatin-xyloglucan) against *Escherichia coli* invasion of intestinal epithelial cells: results of an *in vitro* study. Future Microbiol. 11 (5), 651–658. 10.2217/fmb-2016-0022 27022857

[B14] De Seta F. Caruso S. Di Lorenzo G. Romano F. Mirandola M. Nappi R. E. (2021). Efficacy and safety of a new vaginal gel for the treatment of symptoms associated with vulvovaginal atrophy in postmenopausal women: a double-blind randomized placebo-controlled study. Maturitas 147, 34–40. 10.1016/j.maturitas.2021.03.002 33832645

[B15] Ekin M. Yaşar L. Savan K. Temur M. Uhri M. Gencer I. (2011). The comparison of hyaluronic acid vaginal tablets with estradiol vaginal tablets in the treatment of atrophic vaginitis: a randomized controlled trial. Arch. Gynecol. Obstet. 283 (3), 539–543. 10.1007/s00404-010-1382-8 20135132

[B16] Gao Z. Fu R. Li X. Wang J. He Y. (2021). Safety assessment of microbicide 2P23 on the rectal and vaginal microbiota and its antiviral activity on HIV infection. Front. Immunol. 12, 702172. 10.3389/fimmu.2021.702172 34447373 PMC8382973

[B17] Garcia N. Gondran C. Menon G. Mur L. Oberto G. Guerif Y. (2011). Impact of AQP3 inducer treatment on cultured human keratinocytes, *ex vivo* human skin and volunteers: impact of AQP3 inducer treatment. Int. J. Cosm. Sci. 33 (5), 432–442. 10.1111/j.1468-2494.2011.00651.x 21401652

[B18] Glazener C. M. Abdalla M. Stroud P. Naji S. Templeton A. Russell I. T. (1995). Postnatal maternal morbidity: extent, causes, prevention and treatment. Br. J. Obstet. Gynaecol. 102 (4), 282–287. 10.1111/j.1471-0528.1995.tb09132.x 7612509

[B19] Guo L. Chen H. Li Y. Zhou Q. Sui Y. (2013). An aquaporin 3-notch1 axis in keratinocyte differentiation and inflammation. PLoS ONE 8 (11), e80179. 10.1371/journal.pone.0080179 24260356 PMC3832656

[B20] Hara M. Verkman A. S. (2003). Glycerol replacement corrects defective skin hydration, elasticity, and barrier function in aquaporin-3-deficient mice. Proc. Natl. Acad. Sci. USA. 100 (12), 7360–7365. 10.1073/pnas.1230416100 12771381 PMC165880

[B21] Hara-Chikuma M. Verkman A. S. (2008). Aquaporin-3 facilitates epidermal cell migration and proliferation during wound healing. J. Mol. Med. 86 (2), 221–231. 10.1007/s00109-007-0272-4 17968524

[B22] Huang S. Constant S. De Servi B. Meloni M. Culig J. Bertini M. (2019). *In vitro* safety and performance evaluation of a seawater solution enriched with copper, hyaluronic acid, and eucalyptus for nasal lavage. MDER 12, 399–410. 10.2147/MDER.S209644 PMC6766585 31576180

[B23] Iosif C. S. Bekassy Z. (1984). Prevalence of genito-urinary symptoms in the late menopause. Gynecol. Scand. 63 (3), 257–260. 10.3109/00016348409155509 6730943

[B24] Kandárová H. Bendova H. Letasiova S. Coleman K. P. De Jong W. H. Jírova D. (2018). Evaluation of the medical devices benchmark materials in the controlled human patch testing and in the RhE *in vitro* skin irritation protocol. Toxicol. Vitro. 50, 433–438. 10.1016/j.tiv.2018.02.009 29462660

[B25] Kim S. O. Lee H. S. Ahn K. Park K. (2009). Effect of estrogen deprivation on the expression of aquaporins and nitric oxide synthases in rat vagina. J. Sex. Med. 6 (6), 1579–1586. 10.1111/j.1743-6109.2009.01223.x 19473476

[B26] Kim S. O. Oh K. J. Lee H. S. Ahn K. Kim S. W. Park K. (2011). Expression of aquaporin water channels in the vagina in premenopausal women. J. Sex. Med. 8 (7), 1925–1930. 10.1111/j.1743-6109.2011.02284.x 21492408

[B27] Kunovac Kallak T. (2015). Hormonal regulation of vaginal mucosa. Acta Univ. Ups., 65.

[B28] Leone M. G. (2022). Medical devices made of substances: a new challenge. Front. Drug Saf. Regul. 2, 952013. 10.3389/fdsfr.2022.952013

[B29] Ling J. Wang Y. H. (2023). Association between depressive mood and body image and menopausal symptoms and sexual function in perimenopausal women. World J. Clin. cases. 11 (32), 7761–7769. 10.12998/wjcc.v11.i32.7761 38073680 PMC10698428

[B30] Marletta M. (2020). The new regulation 2017/745: an opportunity for innovation. Pharm. Adv. 01 (01). 10.36118/pharmadvances.01.2020.03s

[B31] Meloni M. Buratti P. Carriero F. Ceriotti L. (2021). *In vitro* modelling of barrier impairment associated with gastro-oesophageal reflux disease (GERD). CEG 14, 361–373. 10.2147/CEG.S325346 PMC8436176 34526798

[B32] Messinger-Rapport B. J. Thacker H. L. (2001). Prevention for the older woman. A practical guide to hormone replacement therapy and urogynecologic health. Geriatrics 56 (9), 32–42. 11582972

[B33] Minkin M. R. Reiter S. (2014). Postmenopausal vaginal atrophy: evaluation of treatment with local estrogen therapy. IJWH 281, 281. 10.2147/ijwh.s57900 PMC3958548 24648772

[B34] Naumova I. Castelo-Branco C. (2018). Current treatment options for postmenopausal vaginal atrophy. IJWH 10, 387–395. 10.2147/IJWH.S158913 PMC6074805 30104904

[B35] Notelovitz M. (1997). Urogenital aging: solutions in clinical practice. Intl. J. Gynecol. and Obste. 59 (S1), S35–S39. 10.1016/s0020-7292(97)90197-1 9386214

[B36] Palma F. Volpe A. Villa P. Cagnacci A. Writing Group of AGATA Study (2016). Vaginal atrophy of women in postmenopause. Results from a multicentric observational study: the AGATA study. Maturitas 83, 40–44. 10.1016/j.maturitas.2015.09.001 26421474

[B37] Park K. Han H. J. Kim S. W. Jung S. I. Kim S. O. Lee H. S. (2008). Expression of aquaporin water channels in rat vagina: potential role in vaginal lubrication. J. Sex. Med. 5 (1), 77–82. 10.1111/j.1743-6109.2007.00650.x 18069995

[B38] Pellegatta G. Spadaccini M. Lamonaca L. Craviotto V. D’Amico F. Ceriotti L. (2020). Evaluation of human esophageal epithelium permeability in presence of different formulations containing hyaluronic acid and chondroitin sulphate. MDER 13, 57–66. 10.2147/MDER.S234810 PMC7069498 32210642

[B39] Pellevoisin C. Bouez C. Cotovio J. (2018a). Cosmetic industry requirements regarding skin models for cosmetic testing. J. Tissue Eng. Regen. Med. Els, 3–37. 10.1016/b978-0-12-810545-0.00001-2

[B40] Pellevoisin C. Videau C. Briotet D. Grégoire C. Tornier C. Alonso A. (2018b). SkinEthic^TM^ RHE for *in vitro* evaluation of skin irritation of medical device extracts. Toxicol. Vitro. 50, 418–425. 10.1016/j.tiv.2018.01.008 29339149

[B41] Pérez-López F. R. Phillips N. Vieira-Baptista P. Cohen-Sacher B. Fialho SCAV Stockdale C. K. (2021b). Management of postmenopausal vulvovaginal atrophy: recommendations of the international society for the study of vulvovaginal disease. Gynecol. Endocrinol. 37 (8), 746–752. 10.1080/09513590.2021.1943346 34169794

[B42] Pérez-López F. R. Vieira-Baptista P. Phillips N. Cohen-Sacher B. Fialho SCAV Stockdale C. K. (2021a). Clinical manifestations and evaluation of postmenopausal vulvovaginal atrophy. Gynecol. Endocrinol. 37 (8), 740–745. 10.1080/09513590.2021.1931100 34036849

[B43] Potter N. Panay N. (2021). Vaginal lubricants and moisturizers: a review into use, efficacy, and safety. Climacteric 24 (1), 19–24. 10.1080/13697137.2020.1820478 32990054

[B44] Pridgeon C. S. Schlott C. Wong M. W. Heringa M. B. Heckel T. Leedale J. (2018). Innovative organotypic *in vitro* models for safety assessment: aligning with regulatory requirements and understanding models of the heart, skin, and liver as paradigms. Arch. Toxicol. 92 (2), 557–569. 10.1007/s00204-018-2152-9 29362863 PMC5818581

[B45] Racchi M. Govoni S. (2020). The concept of non-pharmacological mechanism of action in medical devices made of substances in practice: what pharmacology can do to promote the scientific implementation of the European medical device regulation. Pharm. Adv. 01 (01). 10.36118/pharmadvances.01.2020.02s

[B46] Racchi M. Govoni S. Lucchelli A. Capone L. Giovagnoni E. (2016). Insights into the definition of terms in European medical device regulation. Expert Rev. Med. Devices. 13 (10), 907–917. 10.1080/17434440.2016.1224644 27559622

[B47] Santoro N. Roeca C. Peters B. A. Neal-Perry G. (2021). The menopause transition: signs, symptoms, and management options. J. Clin. Endocrinol. and Metabol. 106 (1), 1–15. 10.1210/clinem/dgaa764 33095879

[B48] Sardi C. Garetto S. Capone L. Galbiati V. Racchi M. Govoni S. (2018). Experimental paradigm for the assessment of the non-pharmacological mechanism of action in medical device classification: the example of glycerine as laxative. Front. Pharmacol. 9, 1410. 10.3389/fphar.2018.01410 30581385 PMC6292988

[B49] Stabile G. Ricci G. Scalia M. S. De Seta F. (2021). Induced dryness stress on human vaginal epithelium: the efficacy of a new vaginal gel. Gels 7 (4), 157. 10.3390/gels7040157 34698175 PMC8544400

[B50] Thornton K. Chervenak J. Neal-Perry G. (2015). Menopause and sexuality. J. Clin. Endocrinol. Metab. 44 (3), 649–661. 10.1016/j.ecl.2015.05.009 PMC5994393 26316248

[B51] Verkman A. S. Anderson M. O. Papadopoulos M. C. (2014). Aquaporins: important but elusive drug targets. Nat. Rev. Drug Discov. 13 (4), 259–277. 10.1038/nrd4226 24625825 PMC4067137

[B52] Wilhite M. (2018). Vaginal dryness. Int. Med. Els., 592–599.e2. 10.1016/b978-0-323-35868-2.00059-1

